# Effects of Body Composition and Anthropometric Profiles on Competitive Performance in U14 Male Basketball Players

**DOI:** 10.3390/sports14060228

**Published:** 2026-06-02

**Authors:** João Rocha, João Serrano, Almudena Martinez-Sanchez, Amália Campos-Redondo, Sergio José Ibáñez

**Affiliations:** 1 Grupo de Optimización del Entrenamiento y Rendimiento Deportivo [GOERD], Universidad de Extremadura, 10003 Cáceres, Spain; amartinepzu@unex.es (A.M.-S.); amaliacr@unex.es (A.C.-R.); sibanez@unex.es (S.J.I.); 2 Sport Physical Activity and Health Research & Innovation Center [SPRINT], Instituto Politécnico de Castelo Branco, 6000-084 Castelo Branco, Portugal; j.serrano@ipcb.pt

**Keywords:** morphological characteristics, lean mass, Performance Index Rating (PIR), youth basketball, talent identification

## Abstract

Body composition and anthropometric characteristics are considered relevant factors in youth basketball performance, yet evidence in early adolescence remains limited. This study aimed to analyze the influence of these characteristics on competitive performance in U14 male basketball players from Portuguese regional selection teams. Ninety-six athletes were assessed during a national youth tournament using a cross-sectional, descriptive, and correlational design. Body composition variables (weight, height, BMI, muscle mass, fat mass, fat-free mass, bone mass, and total body water) were measured using a Tanita MC-780MA bioelectrical impedance analyzer. Competitive performance was evaluated through the Performance Index Rating (PIR), normalized per minute of play. Spearman’s correlations showed moderate positive associations between PIR and height (ρ = 0.296), muscle mass (ρ = 0.280), fat-free mass (ρ = 0.280), bone mass (ρ = 0.274), and total body water (ρ = 0.262). Although multivariable regression analyses did not identify significant individual predictors due to severe multicollinearity, principal component analysis revealed an “overall body size” factor, mainly reflecting lean and bone mass, that significantly predicted PIR (β = 0.046, *p* < 0.001). Physically more developed players tended to demonstrate higher competitive effectiveness. Monitoring body composition may support youth development programs when combined with functional and technical assessments to inform individualized training and talent identification strategies.

## 1. Introduction

Basketball is characterized by intermittent high-intensity efforts, combining periods of moderate activity with explosive movements such as sprints, jumps, rapid changes of direction, and defensive maneuvers [1,2,3]. These actions impose substantial mechanical demands due to frequent accelerations and decelerations, while simultaneously requiring the execution of complex technical and tactical skills [4,5]. Muscular power and anaerobic capacity are therefore critical determinants of basketball performance, supported by both neuromuscular and cardiovascular systems [6,7,8].

Success in basketball is strongly influenced by anthropometric and physiological attributes, which evolve across developmental stages [1,9,10]. During adolescence, particularly between ages 14 and 17, athletes undergo significant changes in body size, composition, and functional capacity [11,12]. Players with advanced biological maturation often display advantages such as greater height, limb length, and strength, which are associated with superior performance outcomes [1,13]. These morphological and maturational differences contribute not only to individual performance but also to talent selection processes at the youth level. Early-maturing athletes are frequently overrepresented in talent pathways because of temporary physical advantages linked to maturation [14]. However, despite this recognition, studies that do not control for biological maturation may overestimate the role of anthropometric characteristics in youth performance.

Body composition plays a particularly relevant role, as lower fat mass and greater lean tissue enhance the execution of explosive and high-intensity actions, while excess body fat can impair performance [15,16]. Previous studies in basketball and other team sports have reported positive associations between fat-free mass and technical performance indicators, including points scored, rebounds, and composite performance indices [12,17]. These findings suggest that structural and compositional factors may condition competitive effectiveness, although evidence remains limited in the Portuguese youth context, particularly at the U14 level.

Despite the growing body of international literature linking anthropometric and physiological attributes to basketball performance [16,18], few studies have used game-specific metrics such as the Performance Index Rating (PIR), which integrates offensive and defensive contributions and is widely used in European competitions [12]. Moreover, existing research has often focused on fitness testing [19], rather than on in-game performance. Recent evidence in professional basketball has also highlighted that body composition evolves throughout the competitive season, showing specific adaptations according to playing position and their relationship with on-court performance [20].

Therefore, the present study aims to analyze the influence of body composition and anthropometric characteristics on competitive performance in U14 male basketball players selected for regional teams in Portugal. Understanding these associations may contribute to talent identification and provide coaches with evidence to design individualized training strategies for adolescent athletes, preventing injuries.

To operationalize this aim, four specific objectives were defined: (a) To describe the biological characteristics of U14 male basketball players participating in the regional selection tournament in Portugal; (b) To analyze the relationship between athletic performance and biological characteristics, specifically focusing on body composition indicators such as lean mass, fat mass, and bone mass, and their association with performance measured through the PIR; (c) To predict performance based on biological characteristics, identifying which body composition variables serve as the best predictors of PIR among the participants; and (d) To identify and verify possible combinations of body composition variables that best explain PIR, exploring whether specific body composition patterns are associated with higher performance, and whether distinct athlete profiles can be characterized in terms of body composition and performance outcomes.

## 2. Material and Methods

### 2.1. Study Design

This research followed a quantitative approach, adopting a cross-sectional, descriptive, and correlational design [21,22,23]. The primary aim was to examine the relationship between body composition, anthropometric indicators, and in-game performance in adolescent basketball players.

### 2.2. Participants

The sample comprised 96 male athletes (12 players × 8 teams) competing in Group A of the Portuguese U14 National Regional Selections Tournament. Data was collected during the qualifying phase for the national finals, ensuring ecological validity by evaluating athletes under real competitive conditions.

### 2.3. Variables and Instruments

In this study, two types of variables were considered: the dependent variable, represented by game performance, and the independent variables, corresponding to anthropometric and body composition indicators.

The dependent variable was the PIR, a composite indicator widely used in basketball to quantify competitive effectiveness. PIR integrates offensive and defensive contributions such as points scored, rebounds, assists, steals, and turnovers. To ensure comparability across athletes, PIR was normalized per minute of play.

The PIR was calculated using the following formula:

PIR = (Points + Rebounds + Assists + Steals + Blocks) − (Missed Field Goals + Missed Free Throws + Turnovers)

The independent variables included eight anthropometric and body composition indicators: body weight (kg), height (cm), body mass index (kg·m^−2^), muscle mass (kg), fat mass (kg), fat-free mass (kg), bone mass (kg), and total body water (L). These indicators were selected based on their relevance in previous research analyzing the physical and morphological determinants of basketball performance in youth athletes.

These variables were selected based on their relevance in previous research examining the physical profiles and performance outcomes of youth basketball players [12,16,24]. The inclusion of both structural and compositional indicators allowed for a comprehensive analysis of the potential influence of biological characteristics on athletic performance.

Anthropometric and body composition data were collected using validated and standardized equipment to ensure measurement accuracy and consistency across participants. The assessments included body weight, height, body mass index (BMI), muscle mass, fat mass, fat-free mass (FFM), bone mass (BM), and total body water (TBW). Body composition was assessed using a multi-frequency segmental bioelectrical impedance analyzer (Tanita MC-780MA, Tanita Corp., Tokyo, Japan), a professional segmental bioelectrical impedance analyzer (BIA). This device operates with multifrequency technology (5 kHz, 50 kHz, and 250 kHz), allowing for precise differentiation between intracellular and extracellular water, and provides a segmental analysis of the body (arms, legs, and trunk). The Tanita is medically certified (CE MDD Class IIa, NAWI Class III) and suitable for use in populations aged 5 to 99 years [25].

Performance was evaluated using the PIR, a composite metric widely used in basketball to quantify individual contributions during gameplay. PIR includes variables such as points scored, rebounds, assists, steals, and turnovers. To account for differences in playing time, PIR was normalized per minute, allowing for standardized comparisons across athletes [12,16].

### 2.4. Procedures

To initiate the data collection process, the research team formally contacted the Portuguese Basketball Federation, which facilitated access to the regional U14 selections and authorized the implementation of the study. Upon approval of the research protocol by the Institutional Bioethics Committee (approval nº 113/2024), each regional team was contacted and provided with detailed information about the study objectives and procedures. Informed consent forms were distributed to the legal guardians of all participants, and assent was obtained from the athletes themselves, in accordance with ethical standards.

Once participation was confirmed, the research team prepared all necessary equipment for data collection. All measurements were conducted on game day, under standardized conditions to ensure consistency and ecological validity. Athletes were assessed in the morning prior to competition under standardized conditions. As all participants were U14 children sharing the same environment (including accommodation, meals, and daily activities), no additional pre-assessment instructions were required. Measurements were conducted in a controlled, non-fasted state. Athletes were evaluated barefoot and wearing light sports clothing to minimize external influences on body composition outcomes.

Anthropometric and body composition variables were measured using validated instruments and standardized protocols, ensuring methodological rigor and reliability of the data, given that all participants were U14 children sharing the same environment, hydration status may have had a minor influence on body composition estimates, representing a small methodological limitation. This approach ensured that data collection was both ethically sound and scientifically robust, allowing for accurate analysis of the relationship between body composition and performance in adolescent basketball players.

### 2.5. Statistical Analysis

Descriptive statistics for continuous variables are presented as mean ± standard deviation (SD), as well as median and interquartile range (IQR), to provide robust estimates for variables that deviated from normality. The Shapiro–Wilk test was used to assess the normality of the data distributions. As most variables violated the assumption of normality (*p* < 0.05), Spearman’s rank-order correlation was employed to examine associations between the PIR and body composition variables, including weight, height, BMI, muscle mass, fat mass, fat-free mass, bone mass, and total body water.

Correlation analysis was guided by a framework [26] that distinguishes between Pearson’s product-moment correlation coefficient (appropriate for normally distributed, continuous data) and Spearman’s rank correlation, which is more robust for non-normal, ordinal, or outlier-prone data. Both coefficients range from −1 to +1, with values closer to ±1 indicating stronger associations. Specifically, correlations were interpreted as follows: trivial (r < 0.1), low (0.1 ≤ r < 0.3), moderate (0.3 ≤ r < 0.5), moderately high (0.5 ≤ r < 0.7), high (0.7 ≤ r < 0.9), and nearly perfect (r ≥ 0.9).

To identify predictors of performance, a multivariable linear regression was initially performed using body composition variables as independent predictors of PIR. However, variance inflation factors (VIFs) revealed severe multicollinearity among predictors, which compromised the interpretability of individual coefficients.

Given the severe multicollinearity observed among anthropometric and body composition variables, unsupervised principal component analysis (PCA) was adopted for the eight body composition variables as the main multivariate approach to reduce dimensionality and obtain more interpretable predictors of competitive performance. Prior to PCA, all variables were centered and scaled. The first two principal components (PC1 and PC2) were retained, accounting for over 96% of the cumulative variance. A linear regression model was then fitted using ordinary least squares (OLS), with heteroskedasticity-consistent standard errors (HC3) to ensure robust inference.

Model outputs included regression coefficients, 95% confidence intervals, and two-tailed *p*-values, with statistical significance set at α = 0.05. Data analysis was conducted using RStudio (version 2025.05.1; Posit Software, Boston, MA, USA).

### 2.6. Ethics

Participation was voluntary, and informed consent was obtained from both athletes and their legal guardians. The study protocol received ethical approval from the University Bioethics Committee (approval nº 113/2024) and complied with the principles of the Declaration of Helsinki [27], ensuring the protection of participants’ rights, privacy, and well-being throughout the research process.

## 3. Results

Table 1 presents the descriptive statistics for all study variables. Values are presented as mean ± standard deviation (SD), together with median and interquartile range (IQR: Q1–Q3), due to non-normal data distribution. The mean performance index ratio (PIR) was 0.24 (0.45), with a Shapiro–Wilk statistic of W = 0.991 (*p* = 0.088), indicating approximate normality. In contrast, the anthropometric and body-composition measures (weight (60.79 ± 12.63 kg), height (174.89 ± 10.88 cm), BMI (19.73 ± 2.68 kg·m^−2^), muscle mass (MM; 52.46 ± 9.13 kg), fat mass (FM; 5.56 ± 4.62 kg), fat-free mass (FFM; 55.24 ± 9.57 kg), bone mass (BM; 2.78 ± 0.44 kg) and total body water (TBW; 41.05 ± 6.21 L)) all yielded W < 0.99 and *p* < 0.05, reflecting significant departures from normality. These results justified the use of non-parametric correlation and robust regression techniques in subsequent analyses.

Spearman’s correlations between PIR and body composition indicators are summarized in Table 2. PIR was moderately and positively associated with height (ρ = 0.296, *p* < 0.001), MM (ρ = 0.280, *p* < 0.001) and FFM (ρ = 0.280, *p* < 0.001), as well as with weight (ρ = 0.272, *p* < 0.001) and BM (ρ = 0.274, *p* < 0.001). TBW also showed a modest positive correlation (ρ = 0.262, *p* < 0.001). Smaller but still significant relationships were observed for FM (ρ = 0.158, *p* = 0.008) and BMI (ρ = 0.140, *p* = 0.017). All associations reached statistical significance (*p* < 0.05), justifying further multivariable modelling.

The multivariable regression model did not identify significant predictors of PIR (Supplementary Table S1). This result may be partially explained by the relative homogeneity of the sample, which limited variability among players and reduced statistical power. However, the primary issue was the presence of extreme multicollinearity among predictors, as indicated by very high variance inflation factors (VIFs). These results suggest that the included variables represent overlapping constructs related to overall body size, limiting the interpretability of individual regression coefficients.

**Figure 1:**
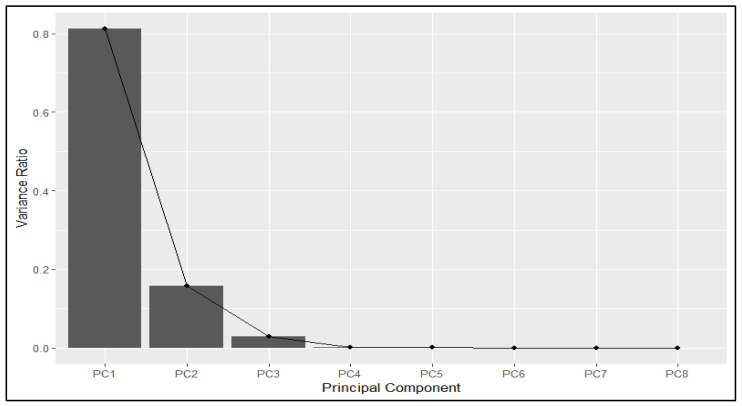
Results of the Principal Component Analysis (PCA) of the influence of body composition variables in Performance Index Ratio (PIR).

A principal-component analysis was performed on eight body-composition variables (weight, height, BMI, muscle mass, fat mass, fat-free mass, bone mass and total body water), after centering and scaling the data (see Figure 1). The variance explained by each principal component is illustrated by the curve in the scree plot, with the first principal component (PC1) accounted for 81.2% and the second (PC2) for 15.7% of the total variance. The bar chart represents the variable loadings on each component. PC1 exhibited uniformly positive loadings across all variables (0.263–0.389), representing a “overall body size” factor (lean mass, bone mass and weight, with a lesser contribution from fat mass). The second component (PC2) explained a further 15.7% of the variance (96.9% cumulatively), loading positively on BMI (0.560) and fat mass (0.608) and negatively on height (−0.471) and lean-mass/total-body-water measures (−0.120 to −0.155), and was interpreted as a “fat versus lean” axis.

**Figure 2:**
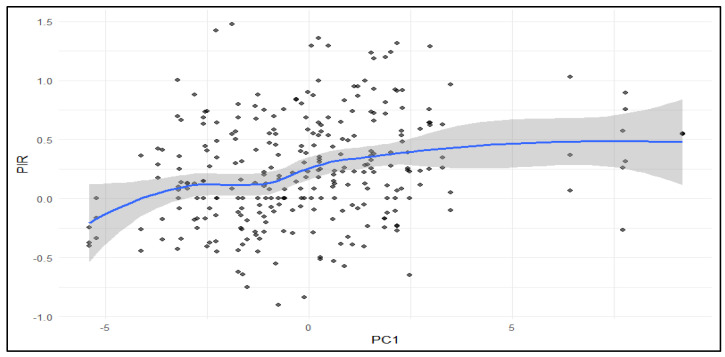
Results for Linear Regression of Performance Index Ratio (PIR) and the overall body size of the players. The solid line represents the fitted linear regression line, and the shaded area indicates the 95% confidence interval.

Subsequently, a linear regression of PIR on PC1 and PC2 was fitted. PC1 emerged as a highly significant predictor (β = 0.046, SE = 0.010; t = 4.58; *p* < 0.001), indicating that a one-unit increase in this component, equivalent to one standard deviation in overall body size, was associated with a 0.046-unit rise in the performance index (see Figure 2). PC2 showed a negative trend (β = −0.041, SE = 0.023; t = −1.78; *p* = 0.077), suggesting that a higher relative fat content, as opposed to lean mass, may slightly reduce performance, although this effect did not reach conventional significance. Together, the two components explained 7.8% of the variance in PIR (R^2^ = 0.078, F2, 284 = 12.05; *p* < 0.001).

## 4. Discussion

The main objective of this study was to understand the relationship between body composition and competitive performance in Portuguese male basketball players in the Under-14 category. The most relevant results point to moderate and positive correlations between the performance index (PIR) and variables such as height, muscle mass, fat-free mass, bone mass and total body water. However, multivariate regression analysis did not reveal significant predictors due to the strong multicollinearity between body indicators. Principal component analysis allowed for the identification of a factor related to body size, which proved to be a relevant predictor of performance. Also, it is important to note that basketball performance in youth athletes is inherently multifactorial, involving not only physical and anthropometric factors, but also technical skills, tactical understanding, and psychological attributes. The absence of these variables should be considered when interpreting the present findings.

The descriptive values obtained are in line with previous data on young Portuguese athletes [12]. The players in this study had slightly higher height and body mass, which may indicate a positive trend in physical development in this age group. Similar patterns were observed in players of the Chilean U-14 national team, who demonstrated greater stature and body mass compared to general population patterns [28]. Also, in elite Spanish athletes, an association was identified between early biological maturation, greater fat-free mass and better performance indicators, such as speed and thrust [16]. Despite these similarities, Portuguese players continue to present lower values in height and lean mass when compared to Spanish players of the same age, which reinforces the existence of anthropometric differences between countries [29]. These variations may reflect differences in talent identification programs, training volume and rhythm of maturation, directly influencing the physical profiles of young athletes.

Correlational analysis reinforces the role of lean tissue in competitive performance, in line with studies highlighting the contribution of muscle mass and fat-free mass to strength and power actions [12,24]. Other studies have identified significant gains in trunk power and lower limb explosiveness in athletes between the ages of 14 and 18, especially in those with greater muscle mass and lower fat percentage [1]. Similar results were observed in Chile selected players, who had a meso-ectomorphic somatotype and larger lean tissue, associated with better performance compared to non-selected ones [28]. The data obtained also corroborate the negative influence of body fat on actions such as agility and explosiveness [15,16]. In professional players, longitudinal studies have shown stability in body composition throughout the season, although there are positional differences that correlate with performance indicators such as offensive rebounds [20]. Although fat mass has shown a modest association with PIR, a higher fat-to-lean mass ratio (PC2) tends to reduce performance, underlining the importance of optimal body composition. Additionally, studies indicate that the years since peak growth velocity (YAPHV) are a stronger predictor of PIR than anthropometric variables alone [16], highlighting the relevance of biological maturation in the competitive path of young athletes. In this regard, the absence of direct maturation-related indicators in the present study prevents differentiating whether the observed performance advantages were primarily associated with body composition itself or with underlying maturational status.

Regression did not identify significant predictors, mainly due to the multicollinearity between body variables. This methodological limitation of the regression model should also be interpreted in light of the characteristics of the sample. The relatively homogeneous nature of the participants, all selected players competing at a similar performance level, may have reduced inter-individual variability and masked potential relationships. Additionally, the strong intercorrelations among anthropometric and body composition variables resulted in substantial multicollinearity, which prevented the identification of independent predictors. This methodological limitation makes it difficult to distinguish the individual impact of variables such as weight, muscle mass, or fat, given their high degree of interrelation. Previous studies also point out that functional tests, such as agility or repeated sprint ability, are better predictors of in-game performance than anthropometric measures [15,16]. The strong correlation between morphological data limits the explanatory power of regression models (Hernandez-Martinez et al., 2024), with functional tests such as vertical jump, sprint speed, and handgrip strength revealing more robust associations with specific performance in basketball [1]. These findings reinforce the importance of using dimensionality-reduction approaches, such as principal component analysis (PCA), when analyzing closely related biological variables in youth athletes.

Principal component analysis confirmed that overall body size, especially determined by lean and bone mass, is positively related to performance. This observation is in line with evidence associating fat-free mass with greater anaerobic capacity and explosive actions such as jumps and sprints [6,24,30]. Players with greater physical development tend to perform better in situations of contact, rebounds and positioning under the basket, contributing directly to the PIR calculation. In a team sport such as basketball, the results obtained underline the importance of considering methodological limitations in the definition of individualized training strategies and in the physical development of athletes in a team context. Body composition and mechanical profiles must be taken into account to properly adjust the stimuli and competitive loads, promoting a more effective preparation of young players [9].

## 5. Conclusions

This study examined how body composition and anthropometric data relate to competitive performance in Portuguese U14 male basketball players. The results revealed moderate positive correlations between PIR and variables such as height, lean mass, bone mass, and total body water, which indicate that more physically developed athletes tend to perform better. Principal component analysis supported this by identifying an “overall body size” factor, were lean and bone mass are a significant predictor of performance. However, multivariable regression did not yield significant individual predictors due to multicollinearity among the variables. Furthermore, the relative homogeneity of the sample may have limited the variability required to detect meaningful associations between variables. This, combined with the strong multicollinearity observed among body composition indicators, reduced the effectiveness of traditional regression approaches.

From an applied standpoint, these findings underscore the value of monitoring body composition in youth basketball development. While selection should not rely solely on anthropometric criteria, increasing lean mass appears particularly important for explosive movements, physical contact, and rebounding. Coaches and practitioners are encouraged to integrate morphological tracking with functional and technical assessments, allowing for tailored training strategies and more holistic talent identification. Moreover, longitudinal monitoring may offer deeper insights into how changes in body structure and physical capacity shape performance trajectories throughout adolescence.

This study presents several limitations that should be considered when interpreting the findings. First, its cross-sectional design restricts causal inferences regarding the relationship between body composition and performance, and longitudinal approaches would be more appropriate to capture developmental changes over time. Second, the relatively homogeneous sample, consisting of players from Portuguese regional selection teams competing at a similar performance level, may have reduced inter-individual variability and limited the detection of meaningful associations. This aspect, combined with the strong intercorrelations among anthropometric and body composition variables, resulted in substantial multicollinearity, reducing the interpretability of traditional regression models and supporting the use of alternative approaches such as principal component analysis. Additionally, the sample was limited to Portuguese U14 male players, which constrains the generalizability of the findings to other populations, including female athletes and different competitive contexts. Finally, although the PIR is widely used, it does not capture the full multidimensional nature of basketball performance, particularly technical, tactical, and psychological aspects.

An additional limitation of this study is the absence of direct measures of biological maturation, such as maturity offset or years from peak height velocity (YAPHV). Considering that U14 players are undergoing substantial maturational changes during adolescence, differences in biological age may partially explain variations in anthropometric characteristics and competitive performance. Consequently, the associations observed between body composition and PIR should be interpreted cautiously, as more biologically mature players may present physical advantages independent of training status or technical ability.

## Figures and Tables

**Table 1:** Descriptive analysis of the variables of the study.

Variable	Mean (SD)	Median (IQR)	Min–Max	W	*p*-Value
PIR (a.u.)	0.24 (0.45)	0.22 (−0.08–0.55)	−0.90–1.48	0.991	0.088
Weight (kg)	60.79 (12.63)	59.70 (52.40–67.40)	35.9–114	0.905	<0.001 *
Height (m)	174.89 (10.88)	175.00 (169.00–182.00)	147–206	0.982	0.001 *
BMI (kg·m^−2^)	19.73 (2.68)	19.50 (18.10–20.90)	13.8–34.4	0.819	<0.001 *
MM (kg)	52.46 (9.13)	52.70 (46.70–57.50)	33.0–79.2	0.968	<0.001 *
FM (kg)	5.56 (4.62)	4.80 (2.70–6.50)	1.10–33.5	0.708	<0.001 *
FFM (kg)	55.24 (9.57)	55.50 (49.20–60.50)	34.8–83.3	0.968	<0.001 *
BM (kg)	2.78 (0.44)	2.80 (2.50–3.00)	1.80–4.10	0.965	<0.001 *
TBW (L)	41.05 (6.21)	41.00 (36.80–44.60)	27.8–59.9	0.959	<0.001 *

SD: standard deviation; IQR: interquartile range (Q1–Q3); W: Shapiro–Wilk estimation; PIR: performance index ratio; BMI: body mass index; MM: muscular mass; FM: fat mass; FFM: free-fat mass; BM: bone mass; TBW: total body water; * *p*-value < 0.05.

**Table 2:** Correlation between Performance Index Ratio (PIR) and body composition variables.

Variable	*ρ*	*p*-Value
Weight (kg)	0.272	<0.001 *
Height (m)	0.296	<0.001 *
BMI (kg·m^−2^)	0.140	0.017 *
MM (kg)	0.280	<0.001 *
FM (kg)	0.158	0.008 *
FFM (kg)	0.280	<0.001 *
BM (kg)	0.274	<0.001 *
TBW (L)	0.262	<0.001 *

*ρ*: Spearman estimation; BMI: body mass index; MM: muscular mass; FM: fat mass; FFM: free-fat mass; BM: bone mass; TBW: total body water; * statistically significant (*p* < 0.05).

## Data Availability

The data presented in this study are available on request from the corresponding author. The data are not publicly available due to the Organic Law 3/2018 of 5 December on the Protection of Personal Data and Guarantee of Digital Rights by the Government of Spain, which requires that this information be kept confidential.

## Supplementary Materials

The following supporting information can be downloaded at: https://www.mdpi.com/article/10.3390/sports14060228/s1, Table S1: Multivariable Linear Regression between Performance Index Ration and body composition variables.

## References

[1] Calleja-González J., Mielgo-Ayuso J., Lekue J.A., Leibar X., Erauzkin J., Jukic I., Ostojic S.M., Ponce-González J.G., Fuentes-Azpiroz M., Terrados N. (2018). Anthropometry and performance of top youth international male basketball players in Spanish national academy. Nutr. Hosp..

[2] García F., Castellano J., Reche X., Vázquez-Guerrero J. (2021). Average Game Physical Demands and the Most Demanding Scenarios of Basketball Competition in Various Age Groups. J. Hum. Kinet..

[3] Scanlan A.T., Wen N., Tucker P.S., Dalbo V.J. (2014). The relation between internal and external training load models during basketball training. J. Strength Cond. Res..

[4] Scanlan A., Dalbo J. (2019). Improving Practice and Performance in Basketball. Sports.

[5] Vázquez-Guerrero J., Suárez-Arrones L., Gómez D.C., Rodas G. (2018). Comparing external total load, acceleration and deceleration outputs in elite basketball players across positions during match play. Kinesiology.

[6] Antoranz Y., Vecino J.D., Durán-Rodríguez H., Jiménez-Reyes P., Jiménez-Saiz S.L. (2025). Correlation among repeated sprint ability, vertical force-velocity profile, and 30-15 IFT performance in elite youth basketball players. E-Balonmano Com J. Sports Sci..

[7] Fox J.L., Scanlan A.T., Stanton R. (2017). A review of player monitoring approaches in basketball: Current trendas and future directions. J. Strength Cond. Res..

[8] Calle O., Mancha-Triguero D., Recio E., Ibáñez S.J. (2025). Physical Fitness Profiling of Youth Basketball Players by Developmental Stage: A Case Study. J. Funct. Morphol. Kinesiol..

[9] Rocha J., Serrano J., López-Sierra P., Ibáñez S.J. (2025). Profiling External Load in U14 Basketball: Cluster Analysis of Competition Performance Using Inertial Devices. Appl. Sci..

[10] Causevic D., Bîca M.D., Hodzic A., Albina A.E., Densley B., Alexe D.I., Zelenovic M., Bichowska-Paweska M., Ibrahimovic M., Savu C.V. (2025). Physical and Performance Characteristics of Elite Youth Male Basketball Players Characterized by Maturity Status. Life.

[11] Lesinski M., Schmelcher A., Herz M., Puta C., Gabriel H., Arampatzis A., Laube G., Büsch D., Granacher U. (2020). Maturation-, age-, and sex-specific anthropometric and physical fitness percentiles of German elite young athletes. PLoS ONE.

[12] Ramos S.A., Massuça L.M., Volossovitch A., Ferreira A.P., Fragoso I. (2021). Morphological and Fitness Attributes of Young Male Portuguese Basketball Players: Normative Values According to Chronological Age and Years from Peak Height Velocity. Front. Sports Act. Living.

[13] Leyhr D., Rösch D., Cumming S.P., Höner O. (2024). Selection-Dependent Differences in Youth Elite Basketball Players’ Relative Age, Maturation-Related Characteristics, and Motor Performance. Res. Q. Exerc. Sport.

[14] Arede J., Fernandes J., Moran J., Norris J., Leite N. (2021). Maturity timing and performance in a youth national basketball team: Do early-maturing players dominate?. Int. J. Sports Sci. Coach..

[15] Gil S.M., Gil J., Ruiz F., Irazusta A., Irazusta J. (2007). Physiological and anthropometric characteristics of young soccer players according to their playing position: Relevance for the selection process. J. Strength Cond. Res..

[16] Torres-Unda J., Zarrazquin I., Gravina L., Zubero J., Seco J., Gil S.M., Gil J., Irazusta J. (2016). Basketball Performance Is Related to Maturity and Relative Age in Elite Adolescent Players. J. Strength Cond. Res..

[17] Hernandez-Martinez J., Perez-Carcamo J., Coñapi-Union B., Canales-Canales S., Negron-Molina M., Avila-Valencia S., Cid-Calfucura I., Herrera-Valenzuela T., Cisterna D., Branco B.H.M. (2024). Relationship between Body Composition and Physical Performance by Sex in Professional Basketball Players. Appl. Sci..

[18] Garcia-Gil M., Torres-Unda J., Esain I., Duñabeitia I., Gil S.M., Gil J., Irazusta J. (2018). Anthropometric parameters, age, and agility as performance predictors in elite female basketball players. J. Strength Cond. Res..

[19] Goniotaki A., Bourdas D.I., Travlos A.K., Bakirtzoglou P., Theos A., Zacharakis E. (2026). Physical and Performance Profiles Differentiate Competitive Levels in U-18 Basketball Players. Sports.

[20] Lopez-Sierra P., Reina M., Lopez-Araya S., Ibáñez S.J. (2025). Impact of training on body composition in elite basketball players. E-Balonmano Com J. Sports Sci..

[21] Montero I., León O.G. (2005). A classification system for method within research reports in Psychology. Int. J. Clin. Health Psychol..

[22] Thomas J.R., Martin P., Etnier J.L., Silverman S.J. (2015). Research Methods in Physical Activity.

[23] Ibáñez S.J., Feu S. (2026). Research designs in Sports Science. E-Balonmano Com J. Sport Sci..

[24] Correas-Gomez L., Bentíez-Flores S., Calleja-Gonzláez J., Carnero E.A. (2023). Quality of lean body mass and jump capacity in high performance young basketball players lean body mass and jump capacity. J. Sports Sci..

[25] Kelly J., Metcalfe J. (2012). Validity and Reliability of Body Composition Analysis Using the Tanita BC418-MA. J. Exerc. Physiol..

[26] Schober P., Boer C., Schwarte L.A. (2018). Correlation Coefficients: Appropriate Use and Interpretation. Anesth. Analg..

[27] World Medical Association (2025). World Medical Association Declaration of Helsinki: Ethical Principles for Medical Research Involving Human Participants. Jama-J. Am. Med. Assoc..

[28] Gajardo-Burgos R., Barría-Vargas C., Flández-Valderrama J., Avendaño-Chipón R., Barría-Pailaquilén R.M., Monrroy-Uarac M. (2018). Perfil Antropométrico de Basquetbolistas Sub-14 Chilenos. Int. J. Morphol..

[29] Mancha-Triguero D., García-Rubio J., Antúnez A., Ibáñez S.J. (2020). Physical and Physiological Profiles of Aerobic and Anaerobic Capacities in Young Basketball Players. Int. J. Environ. Res. Public Health.

[30] Ujakovic F., Salazar H., Plesa J., Svilar L. (2024). Elite basketball game external load varies between different teams and competition. Kinesiology.

