# Supplementary material for: Effects of Body Composition and Anthropometric Profiles on Competitive Performance in U14 Male Basketball Players
Source: Sports (Basel). 2026 Jun 2;14(6):228. doi: 10.3390/sports14060228 (PMC13306424; doi:10.3390/sports14060228)
Supplement: Supplementary file 1 [file sports-14-00228-s001.zip › sports-4285092-supplementary.pdf]

Supplementary Table S1. Multivariable Linear Regression between Performance Index Ration and body composition variables.

**Variables**	*B*	***SE* (*OLS*)**	***SE* (*HC3*)**	***IC* 2.5%**	***IC* 97.5%**	***p* (*OLS*)**	***p* (*HC3*)**	***VIF***
Weight	−1.53	0.93	0.93	−3.35	0.29	0.100	0.100	205477.26
Height	0.01	0.02	0.02	−0.03	0.05	0.683	0.683	73.42
BMI	0.02	0.08	0.08	−0.14	0.19	0.776	0.776	73.66
MM	1.63	0.97	0.97	−0.28	3.55	0.094	0.094	118129.09
FM	1.52	0.92	0.92	−0.30	3.34	0.101	0.101	27335.56
TBW	−0.04	0.06	0.06	−0.16	0.08	0.493	0.493	208.36

SE: standard error; OLS: ordinary least square; HC3: heteroskedasticity-consistent; IC: interval coefficients; VIF: variance inflation factor; B: unstandardized regression coefficient representing the expected change in Performance Index Rating;

BMI: body mass index; MM: muscular mass; FM: fat mass; FFM: free-fat mass; BM: bone mass; TBW: total body water; * *p-value* < 0.05.
